# Construction of asthma related competing endogenous RNA network revealed novel long non-coding RNAs and potential new drugs

**DOI:** 10.1186/s12931-019-1257-x

**Published:** 2020-01-10

**Authors:** Yifang Liao, Ping Li, Yanxia Wang, Hong Chen, Shangwei Ning, Dongju Su

**Affiliations:** 1 Department of Respiratory Medicine, The 2nd Affiliated Hospital of Xiamen Medical College, Xiamen, Fujian China; 2 Department of Radiology, The 2nd Affiliated Hospital of Harbin Medical University, Harbin, Heilongjiang China; 3 College of Bioinformatics Science and Technology, Harbin Medical University, Harbin, Heilongjiang China; 4 Department of Respiratory Medicine, The 2nd Affiliated Hospital of Harbin Medical University, Harbin, Heilongjiang China

**Keywords:** Asthma, Long non-coding RNA, mRNA, Competing endogenous RNA network, Drug repositioning

## Abstract

**Background:**

Asthma is a heterogeneous disease characterized by chronic airway inflammation. Long non-coding RNA can act as competing endogenous RNA to mRNA, and play significant role in many diseases. However, there is little known about the profiles of long non-coding RNA and the long non-coding RNA related competing endogenous RNA network in asthma. In current study, we aimed to explore the long non-coding RNA-microRNA-mRNA competing endogenous RNA network in asthma and their potential implications for therapy and prognosis.

**Methods:**

Asthma-related gene expression profiles were downloaded from the Gene Expression Omnibus database, re-annotated with these genes and identified for asthma-associated differentially expressed mRNAs and long non-coding RNAs. The long non-coding RNA-miRNA interaction data and mRNA-miRNA interaction data were downloaded using the starBase database to construct a long non-coding RNA-miRNA-mRNA global competing endogenous RNA network and extract asthma-related differentially expressed competing endogenous RNA network. Finally, functional enrichment analysis and drug repositioning of asthma-associated differentially expressed competing endogenous RNA networks were performed to further identify key long non-coding RNAs and potential therapeutics associated with asthma.

**Results:**

This study constructed an asthma-associated competing endogenous RNA network, determined 5 key long non-coding RNAs (MALAT1, MIR17HG, CASC2, MAGI2-AS3, DAPK1-IT1) and identified 8 potential new drugs (Tamoxifen, Ruxolitinib, Tretinoin, Quercetin, Dasatinib, Levocarnitine, Niflumic Acid, Glyburide).

**Conclusions:**

The results suggested that long non-coding RNA played an important role in asthma, and these novel long non-coding RNAs could be potential therapeutic target and prognostic biomarkers. At the same time, potential new drugs for asthma treatment have been discovered through drug repositioning techniques, providing a new direction for the treatment of asthma.

## Introduction

Asthma is a chronic inflammatory disease of the airway which involves many cells and elements, resulting in airway hyperresponsiveness (AHR), excessive mucous secretion and expiratory airflow obstruction. Patients present with intermittent wheezing, chest tightness, shortness of breath and coughing triggered by infection, exercise, allergens or other stimuli. It is a serious public health problem around the world, affecting individuals of all age [1]. However, it has not been fully studied at the molecular level.

Long non-coding RNA (lncRNA) is a kind of non-coding RNA, with transcripts more than 200 bp in length [2]. In recent years, lncRNA has gained widespread attention, as it can participate in a large range of biological processes, including regulation of apoptosis and invasion, reprogramming stem cell pluripotency, and parental imprinting [3, 4]. Previous studies have revealed some potential lncRNAs in asthma. For instance, a study uncovered that lncRNA TCF7 facilitated human airway smooth muscle cells (ASMCs) growth and migration by targeting TIMMDC1/Akt axis [5]. Meanwhile, Zhang et al. suggested that BCYRN1 may also regulate the proliferation and migration of ASMCs through up-regulation of TRPC1 channel [6]. Another group revealed that lncRNA GAS5/miR-10a/BDNF regulatory axis contributed to the ASMCs proliferation [7]. In terms of asthma immunity, lncRNA MEG3 can regulate RORγt and affect Treg/Th17 balance via inhibiting miR-17 [8].

Competitive endogenous RNA (ceRNA) is a novel regulatory mechanism hypothesis: transcripts such as lncRNA, pseudogene transcripts or mRNA can be used as ceRNAs through microRNA (miRNA) response elements (MREs) to compete with miRNAs to regulate the expression level of the genes, thus affecting the function of the cells [9]. The ceRNA interactions have been found in respiratory diseases, especially in lung cancer. A study of non-small cell lung cancer (NSCLC) showed lncRNA LINC00702 could function as ceRNA for miR-510 to regulate PTEN expression, thus affected the proliferation and metastasis of cancer cells [10]. Another study found lncRNA NR2F2-AS1 promoted NSCLC progression through regulating miR-320b/BMI1 axis [11]. In idiopathic pulmonary fibrosis (IPF), lncRNA PFAR regulated YAP1-Twist axis through targeting miR-138 as ceRNA, affected fibrogenesis in fibrotic lung [12]. The ceRNA mechanism for other respiratory diseases is constantly being explored, but its role in asthma is still unclear.

In this study, based on the ceRNA theory, we aimed to explore the regulatory lncRNA-miRNA-mRNA ceRNA network and key lncRNA in asthma by analyzing gene expression profile using bioinformatic methods. We downloaded the asthma-related gene expression profile (GSE43696) from the Gene Expression Omnibus (GEO) database, re-annotated these genes and identified asthma-specific differentially expressed mRNAs, lncRNAs. We then constructed a lncRNA-miRNA-mRNA global ceRNA network and extracted asthma-related DE ceRNA network, from which we determined 5 key lncRNAs (MALAT1, MIR17HG, CASC2, MAGI2-AS3, DAPK1-IT1). For further understanding of the key lncRNAs, we performed functional enrichment analysis. Additionally, drug repositioning was performed to discover new drug treatments for asthma, we identified 8 potential new drugs (Tamoxifen, Ruxolitinib, Tretinoin, Quercetin, Dasatinib, Levocarnitine, Niflumic Acid, Glyburide).

## Materials and methods

### Gene expression profile

We downloaded the asthma-related gene expression profile from the GEO database (http://www.ncbi.nlm.nih.gov/geo/) with the accession number of GSE43696 [13]. There were 108 samples (20 control and 88 cases). The mRNA sequence data and lncRNA sequence data were downloaded from the GENCODE (http://www.gencodegenes.org/) database. Approval from the Ethics Committee was not required for all the information was obtained from the GEO and GENCODE database.

### Probe re-annotation

#### Sequence alignment

The mRNA sequence, the lncRNA sequence, and the probe sequence were sequence-aligned by the blastn tool, respectively. Basic Local Alignment Search Tool (BLAST) is a set of analysis tools for similarity comparison in protein databases or DNA databases which include blastn, blastp, blastx, tblastn, and tblastx. Blastn is a comparison of a given nucleic acid sequence to a sequence in a nucleic acid database. The length of each probe segment in the probe sequence information is 60, so this data analysis selected the alignment result of the perfect match length of 60.

#### Probe screening

According to the results of the sequence alignment, the probes which completely matched the lncRNA and the probes which completely matched the mRNA were separately screened. Probe screening relied mainly on the following two conditions: (1) The probe is only aligned to the mRNA sequence or only aligned to the lncRNA transcript sequence. (2) The probe is aligned to the only transcript sequence.

### Differential expression analysis

Based on the mRNA-related expression profiles and lncRNA-related expression profiles, the significance analysis of microarrays (SAM) method [14] (q < 0.05, FDR) was used for differential expression analysis, performed using the SAM function in the R package samR [15].

### Network construction

The lncRNA-miRNA interaction data and mRNA-miRNA interaction data were downloaded from the starBase V2.0 database (http://starbase.sysu.edu.cn/starbase2/index.php) [16], the lncRNA-miRNA-mRNA competing endogenous RNA network (global ceRNA network) was constructed by Cytoscape v3.5.1 software [17]. Based on the calculated differentially expressed mRNAs (DE mRNAs) and differentially expressed lncRNAs (DE lncRNAs), combined with global ceRNA network information, a subnetwork related to DE mRNA and DE lncRNA (DE ceRNA network) was extracted.

### Functional enrichment analysis

The following databases were used as the source of function nodes: Gene Ontology (BP,MF,CC) (http://www.geneontology.org/) [18, 19], Kyoto Encyclopedia of Genes and Genomes (KEGG) pathway (https://www.genome.jp/kegg/pathway.html) [20], REACTOME (pathway, reaction) (http://www.reactome.org) [21], Wiki Pathway (https://www.wikipathways.org) [22], InterPro (http://www.ebi.ac.uk/interpro/) [23]. We performed functional enrichment analysis with the adjacent mRNAs of key lncRNAs by the R function ‘phyper’ (*p* < 0.05). And the ‘pheatmap’ function in R package was used for hierarchical clustering analysis, the relative expression levels were shown by the intensity of color.

### Drug repositioning

We obtained the drug-gene targeting relationship and drug annotation information from the DrugBank database (https://www.drugbank.ca/) [24], and downloaded the drug-disease targeting relationship from the Therapeutic Target Database (TTD) (http://bidd.nus.edu.sg/group/cjttd/) [25]. Then, we established a network of drugs and genes, we extracted asthma-related drug-gene interaction networks, integrated with DE ceRNA networks. In this way, we constructed a DE ceRNA-drug network and used Cytoscape v3.5.1 for visualization. To discover new asthma-targeted drugs, we integrated drug and disease targeting information and searched publications for drug repositioning.

## Results

### Construction of global ceRNA network and DE ceRNA networks

We downloaded the asthma related gene expression profile (GSE43696) from the GEO database, with 108 samples (20 control and 88 cases). The mRNA sequence data and lncRNA sequence data were downloaded from the GENCODE database. After downloading the data, we re-annotated the probes, identified DE mRNAs and DE lncRNAs with the method of SAM. As a result, a total of 266 DE mRNAs and 31 DE lncRNAs were identified.

**Fig. 1:**
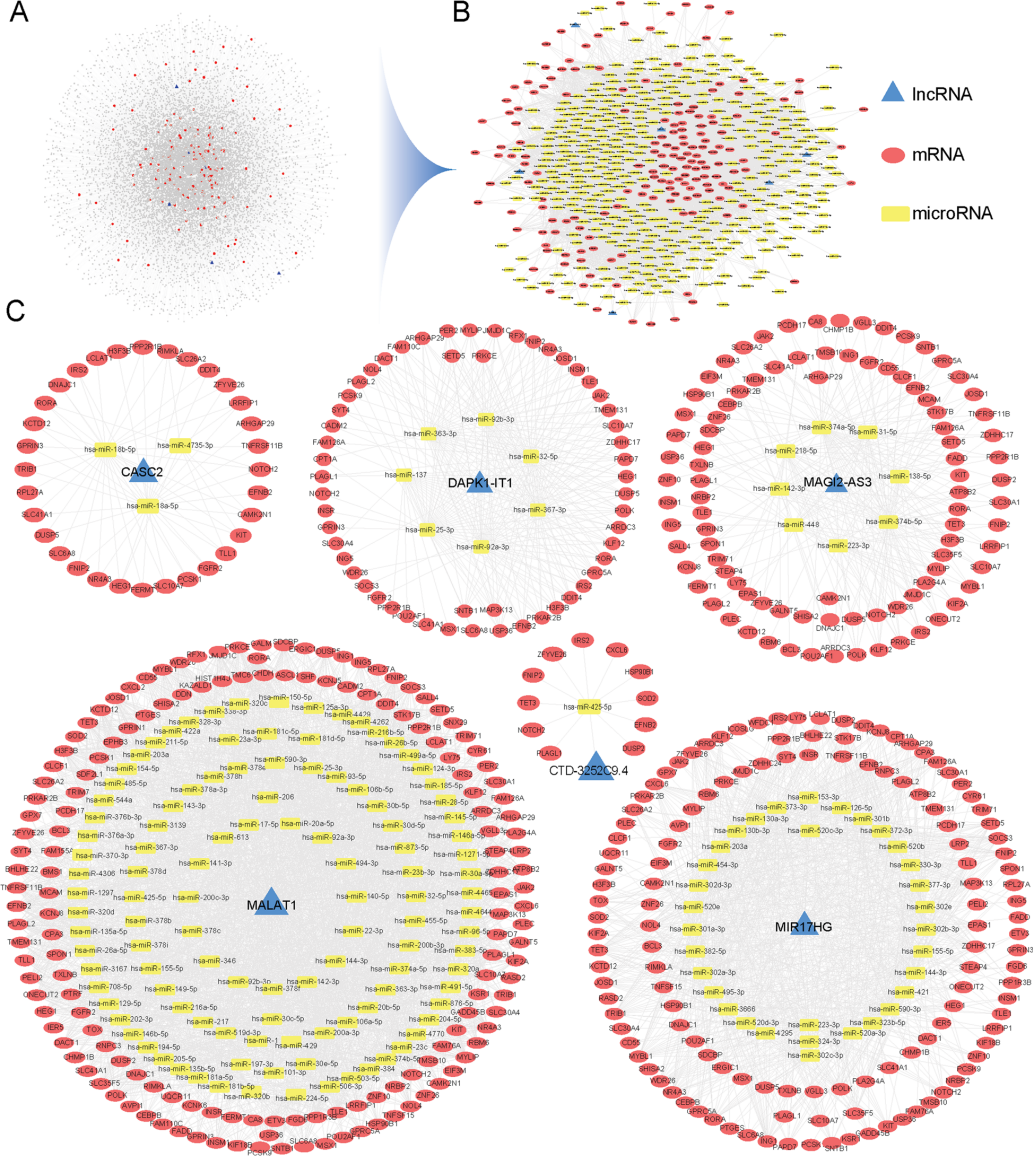
Global ceRNA network and DE ceRNA network. (**a**): Global ceRNA network, 434,187 edges and 15,317 nodes (387 miRNAs, 1128 lncRNAs, 13,802 mRNAs). (**b**): DE ceRNA network, 6800 edges and 547 nodes (378 miRNAs, 6 lncRNAs, and 163 mRNAs). (**c**): 5 key lncRNAs related ceRNA subnetworks. In the above networks, blue triangle = lncRNAs, yellow rectangle = microRNAs, red oval = mRNAs

In this study, lncRNA-miRNA interaction data and mRNA-miRNA interaction data were downloaded from the starBase V2.0 database to construct the global ceRNA network (Fig. 1a), visualization was performed with Cytoscape v3.5.1. There were 434,187 edges and 15,317 nodes in the network, including 387 miRNAs, 1128 lncRNAs, 13,802 mRNAs, 10,212 miRNA-lncRNA interaction pairs (miRNA: 278, lncRNA: 1128), and 423,975 miRNA-mRNA interaction pairs (miRNA: 387, mRNA: 13802). Based on the calculated 266 DE mRNAs and 31 DE lncRNAs, combined with global ceRNA network information, DE ceRNA network was extracted (Fig. 1b). The DE ceRNA network contains 547 points and 6800 sides, including 378 miRNAs, 163 mRNAs, and 6 lncRNAs (MALAT1, MIR17HG, CASC2, MAGI2-AS3, DAPK1-IT1, CTD-3252C9.4). As the lncRNA CTD-3252C9.4 just linked to 1 miRNA, we defined the remaining 5 lncRNAs as the key lncRNAs (MALAT1, MIR17HG, CASC2, MAGI2-AS3, DAPK1-IT1). For more details, we extracted each key lncRNA related ceRNA subnetwork (Fig. 1c).

### Literature validation of RNAs in key lncRNAs related ceRNA subnetworks

**Fig. 2:**
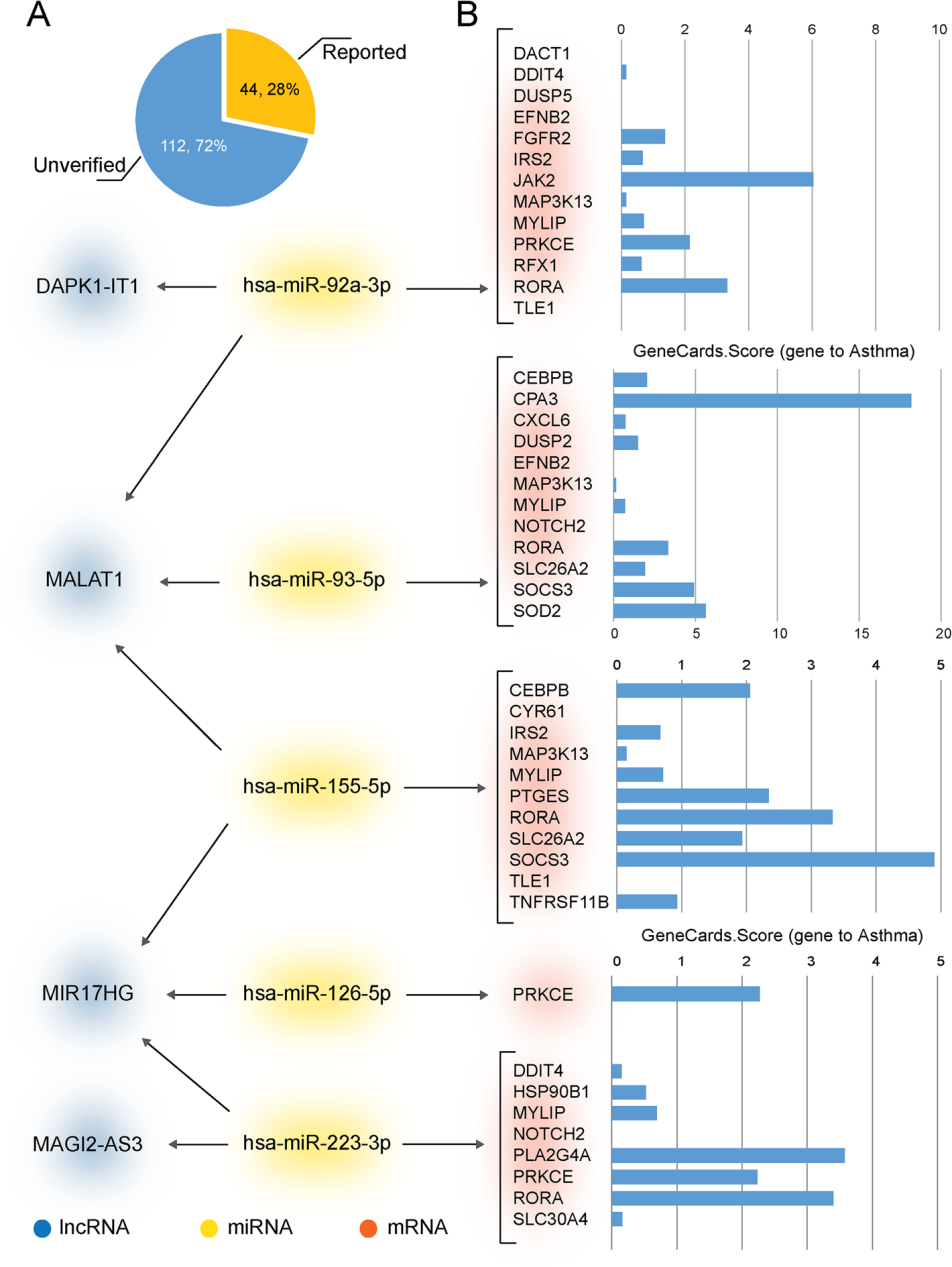
Literature validation on lncRNA, mRNA, and miRNA in key lncRNA related ceRNA subnetworks. (**a**): A total of 156 mRNAs, 44(28%) have been reported to be associated with asthma, 112(72%) were unverified. (**b**): Verified miRNAs linked to the lncRNAs and mRNAs in the ceRNA subnetwork, mRNAs were ranked with scores obtained from the GeneCards database. Blue nodes = lncRNA, yellow nodes = miRNA, red nodes = mRNA

We performed literature validation on lncRNAs, mRNAs, and miRNAs in key lncRNAs related ceRNA subnetworks to determine experimentally confirmed asthma-associated genes. There were 156 mRNAs, 44(28%) have been reported to be associated with asthma, while 112(72%) of them were unverified (Fig. 2a). And we searched GeneCards database for asthma associated mRNAs, there was a score to each mRNA, the higher the score, the closer the mRNA is related to asthma. No study had assessed lncRNA MALAT1, MIR17HG, CASC2, MAGI2-AS3 and DAPK1-IT1 in asthma. As for miRNAs, only 5 miRNAs (hsa-miR-92a-3p, hsa-miR-93-5p, hsa-miR-126-5p, hsa-miR-155-5p, hsa-miR-223-3p) have been verified. We linked the verified miRNAs to lncRNAs and mRNAs that connected to in the ceRNA subnetworks (Fig. 2b). And we considered that if a lncRNA linked to more literature validated miRNAs and mRNAs, it might be closer relevant to asthma.

Among the miRNAs, hsa-miR-92a-3p, hsa-miR-155-5p, and hsa-miR-223-3p were all linked to more than one lncRNA. The regulation of miR-92a-3p modified the transcription of KAT2B and PARP1, which played an important role in the development of combined allergic rhinitis and asthma syndrome [26]. Xiao et al. reported that miR-155 participated in immune regulation by setting a balance between Th1 and Th2 responses, and the expression level of miR-155-5p was highly related to protein expression of the pro-asthmatic Th2 cytokines in bronchoalveolar lavage fluid (BALF) samples [27]. A study of sputum showed miR-223-3p was significantly upregulated in sputum of patients with severe asthma, and was highest in neutrophilic asthma, the expression was also associated with airway obstruction (FEV1/forced vital capacity) [28].

CPA3 got the highest GeneCards score in mRNAs. Dougherty et al. demonstrated that CPA3 expression was especially increased in intraepithelial mast cells from subjects with TH2-high asthma, and it might play a role in asthma through production of angiotensin II [29]. By using the short interfering RNA (siRNA) technology, Zafra MP et al. found that after treated with SOCS3 siRNA, there were significant reduction of GATA3, Foxp3 gene expression and IL-10 mRNA production in eosinophils from asthmatics. In the meantime, SOCS3 siRNA treatment decreased the migration, adhesion and degranulation of eosinophil in response to IL-5. SOCS3 played an important regulatory role in asthma [30]. A metabolomic study of asthmatics indicated that PRKCE might be involved in the sphingolipid metabolism in asthma [31]. The JAK/STAT signaling pathway is an important cytokine signal transduction pathway. Ding F et al. discovered that LPS pretreatment inhibited IL-13 stimulation of ROS generation and the activation of JAK2/STAT6 pathways, thus promoted transcription factor FOXA2, inhibited excessive mucus secretion in asthma [32]. The other group showed miR-375 down regulated the expression of JAK2, inhibited the proliferation and migration of fetal ASM cells through regulating JAK2 / STAT3 signaling [33].

### Functional enrichment analysis revealed key lncRNAs were closely related to asthma

**Fig. 3:**
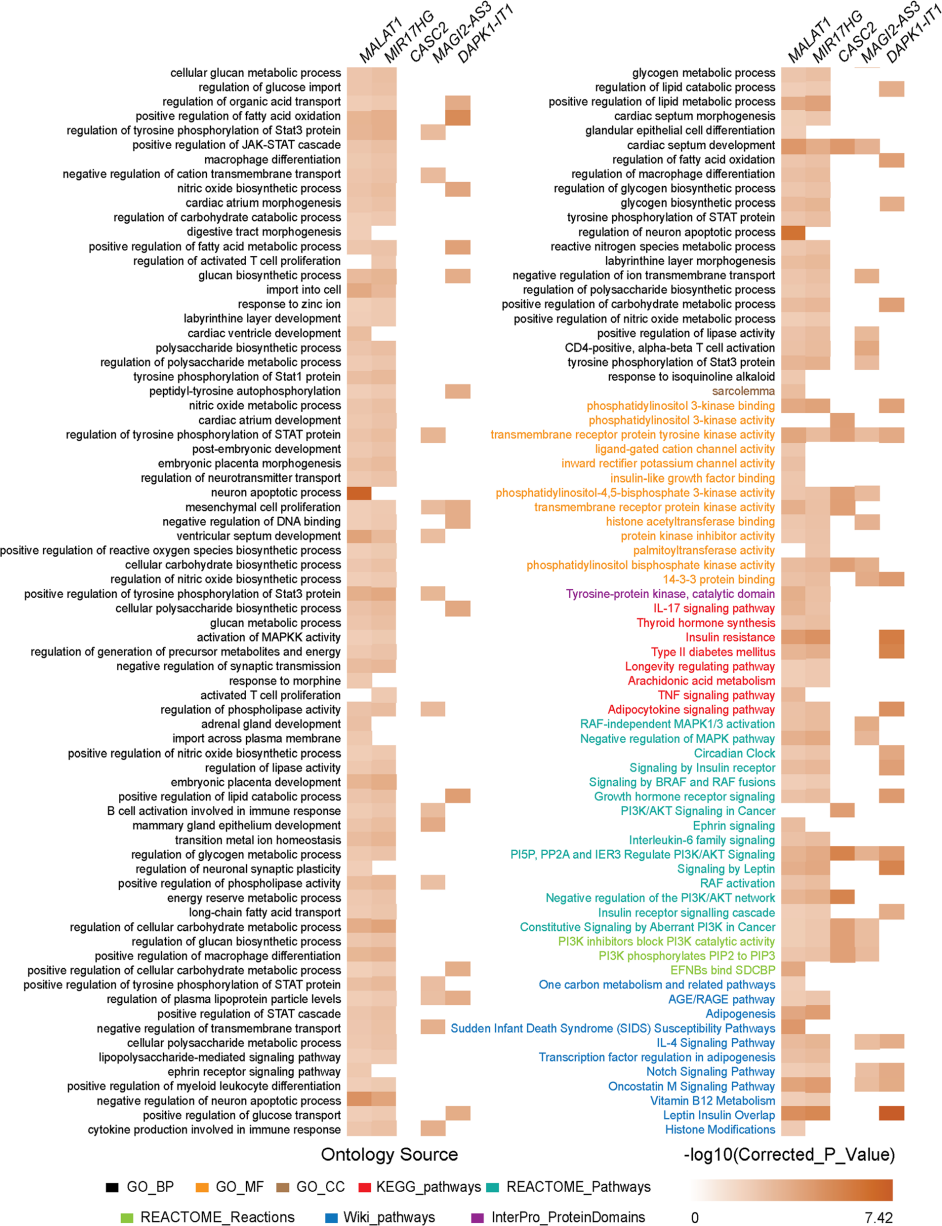
Functional enrichment analysis of 5 key lncRNAs. Texts in different colors were from different ontology source. The relative expression levels were shown by the intensity of color

To better understand the function of these key lncRNAs, functional enrichment analysis of the adjacent mRNAs of key lncRNAs were performed. The results were in eight aspects of the biological process (BP), molecular function (MF), cell composition (CC), KEGG pathways, REACTOME pathways, REACTOME reactions, Wiki pathways and InterPro protein domains (Fig. 3). Among them, MALAT1 and MIR17HG had the richest enrichment results and enriched in almost every aspect.

The functional analysis of MALAT1 revealed 106 GO terms (94 in BP, 1 in CC, 11 in MF), 8 KEGG pathways, 1 InterPro_ProteinDomains, 14 REACTOME_Pathways, 3 REACTOME_Reactions, and 11 Wiki_pathways. For MIR17HG, there were 94 GO terms (85 in BP, 9 in MF), 7 KEGG pathways, 1 InterPro_ProteinDomains, 13 REACTOME_Pathways, 2 REACTOME_Reactions, and 8 Wiki_pathways. MALAT1 and MIR17HG had many common enrichment results that were important in asthma. Such as the regulation of STAT family, “transmembrane receptor protein kinase activity”, “IL-17 signaling pathway”, “Arachidonic acid metabolism”, “Circadian Clock”, “Interleukin-6 family signaling”, etc. Moreover, MIR17HG had been involved in the T cell proliferation, and the component of sarcolemma, while MALAT1 been in TNF signaling pathway. In addition to the other three lncRNAs, CASC2: 6 GO terms (1 in BP, 5 in MF), 4 REACTOME_Pathways, 2 REACTOME_Reactions. MAGI2-AS3: 24 GO terms (19 in BP, 5 in MF), 4 REACTOME_Pathways, 2 REACTOME_Reactions, and 3 Wiki_pathways. DAPK1-IT1: 20 GO terms (17 in BP, 3 in MF), 3 KEGG pathways, 6 REACTOME_Pathways, and 4 Wiki_pathways. Even though CASC2, MAGI2-AS3, and DAPK1-IT1 had fewer results, they were also enriched in many asthma-related functions and pathways, as “cytokine production involved in immune response”, “mesenchymal cell proliferation”, “phosphatidylinositol 3-kinase activity”, etc.

In the biological process, the phosphorylation of STAT took an important role. A study of asthmatic mice model showed after the antigen exposure, phosphorylation of STAT6 and STAT1 occurred at the early stage [34]. The inhibition of STAT1 effectively reduced allergen-induced airway inflammation and AHR [35]. Another study indicated that the activation of STAT3 was higher in mild asthma, and IL-6 stimulation increased STAT3 phosphorylation [36]. In the molecular function, 5 key lncRNAs were all enriched in “transmembrane receptor protein tyrosine kinase activity”. The insulin-like growth factor-I receptor (IGF-IR) is a transmembrane heterotetrameric glycoprotein belonging to the transmembrane tyrosine kinase receptor family, it binds to IGF-I, IGF-II, and insulin [37]. Piñeiro-Hermida et al. investigated that after house dust mite (HDM) exposure, IGF-1R-deficient mice showed absent or attenuated AHR and airway inflammation, compared to control mice, indicated IG-1R as a potential therapeutic target for asthma [38]. IL-17 is an important cytokine in the inflammation of asthma. IL-17 receptor A (IL-17RA) is a receptor subunit of IL-17A and IL-25. IL-17A enhanced airway constriction, and the blockage of IL-17RA, but not IL-17A, induced allergic inflammation and AHR. Therefore, IL-17A pathway can be stopped by blocking IL-17RA [39]. A study of mice showed pre-treatment of HDM allergens/PAR-2 increased IL-17A levels and IL-17RA expression, and the upregulation of IL-17A/IL-17RA deteriorated airway inflammation [40]. As the key lncRNAs involved in so many asthma-related functional results, we deduced that they might be relevant to asthma through gene regulation.

### Drug repositioning to discover new asthma-targeted drugs

**Fig. 4:**
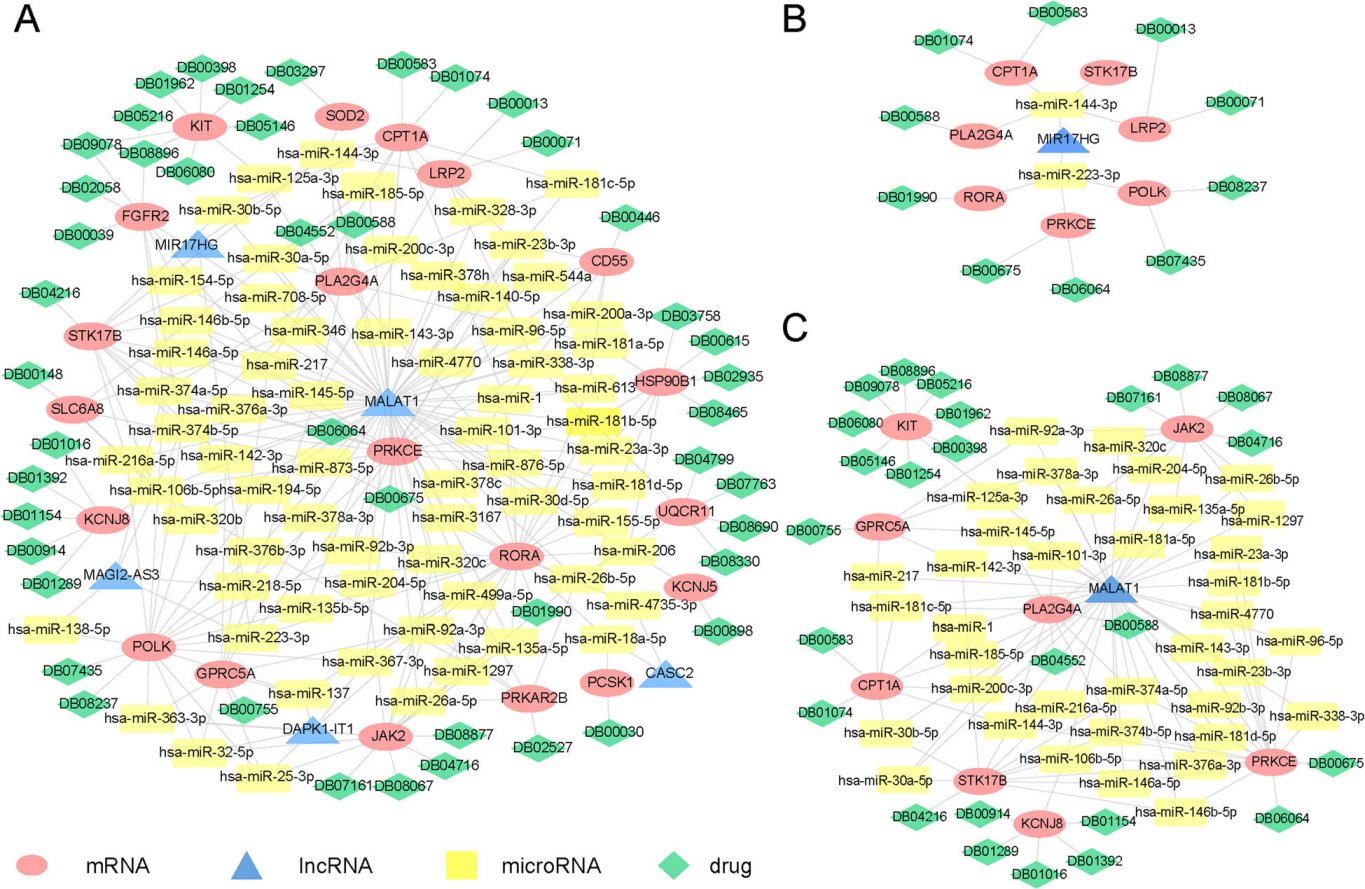
DE ceRNA-drug network and subnetworks. (**a**): DE ceRNA-drug network. (**b**): MIR17HG ceRNA-drug subnetwork. (**c**): MALAT1 ceRNA-drug subnetwork. Red oval = mRNAs, blue triangle = lncRNAs, yellow rectangle = microRNAs, green diamond = drugs

**Fig. 5:**
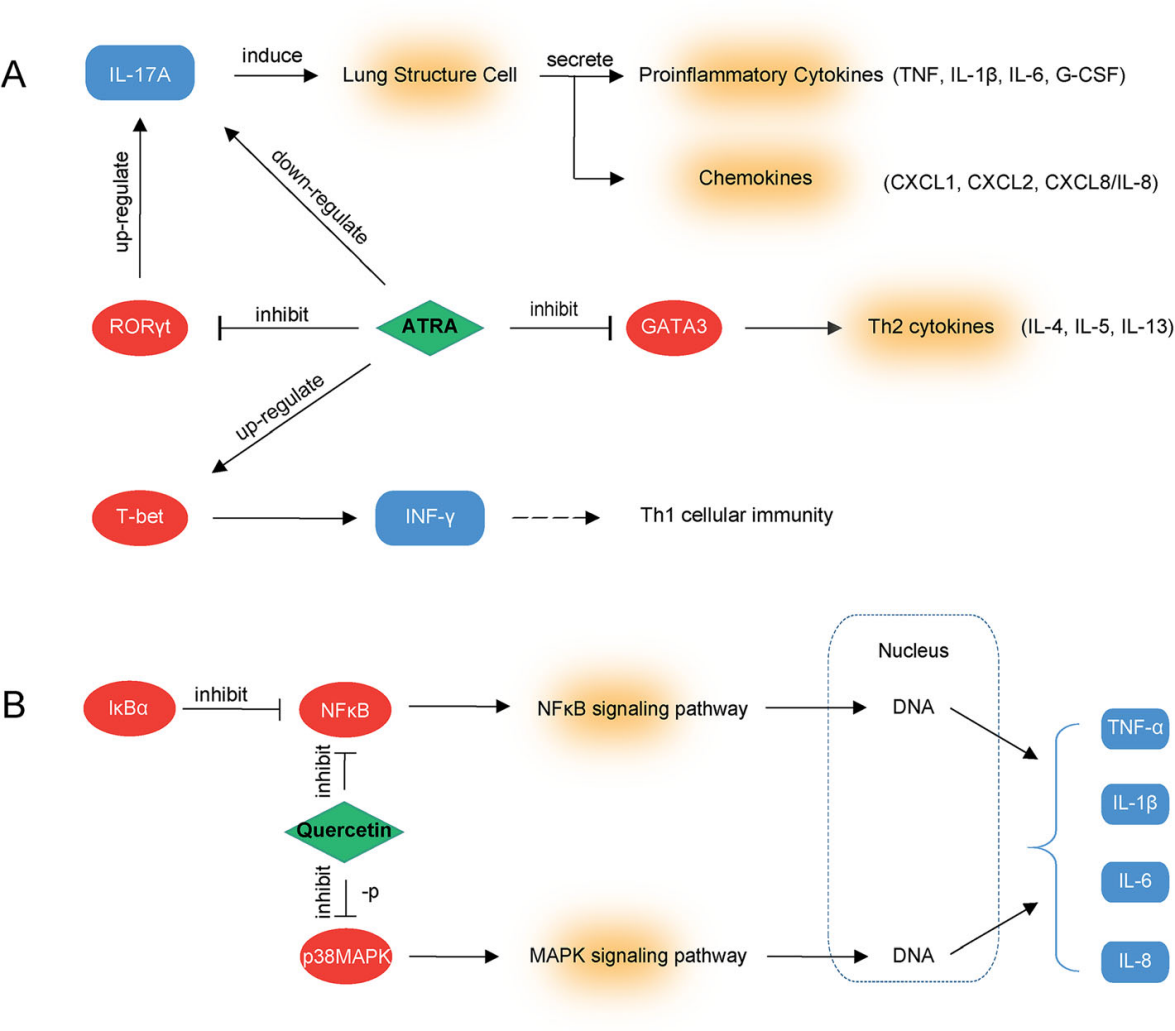
The potential mechanism of drugs in asthma. (**a**): The potential mechanism of Tretinoin (ATRA) in asthma. ATRA decreased Th2 cytokines by inhibiting GATA-3 and up-regulating T-bet expression, also, ATRA inhibited the expression of RORγt, thus decreased the level of IL-17A and the downstream proinflammatory factors. (**b**): The potential mechanism of Quercetin in asthma. Quercetin inhibited gene expression of pro-inflammatory cytokine (TNF-a, IL-1b, IL-6, and IL-8) by regulating the activation of NF-κB and p38 MAPK

Drug repositioning is a strategy to find new indications for drugs which they are not originally intended, it is a faster and cheaper way to develop new therapies. By using the DrugBank and TTD database, we constructed a DE ceRNA-drug network (Fig. 4a), there were 46 drugs that might be the new asthma-targeted drugs. After searching literature in the Pubmed database, 8 drugs were pointed out, they were Tamoxifen, Ruxolitinib, Tretinoin, Quercetin, Dasatinib, Levocarnitine, Niflumic Acid, and Glyburide. In addition to Quercetin and Niflumic Acid which were still under experimenting, the other drugs have been approved by the FDA for disease treatment, and their targeted diseases were not asthma. The detailed information of these drugs was provided in Table 1. And drugs with potential pathways in asthma treatment are expressed in Fig. 5. LncRNA MIR17HG and MALAT1 were associated with all the above 8 drugs, we defined they were closely related with asthma, and extracted the ceRNA-drug subnetwork of MIR17HG and MALAT1 (Fig. 4b and c).

**Table 1:** Detailed information of the new asthma-targeted drugs

DrugBank ID	Name	Gene	Drug Groups	Formula	Disease	Gene Linked lncRNA
DB00675	Tamoxifen	PRKCE	approved	C26H29NO	Breast cancer	MIR17HG MALAT1 MAGI2-AS3 DAPK1-IT1
DB08877	Ruxolitinib	JAK2	approved	C17H18N6	Essential thrombocythemia	MIR17HG MALAT1 MAGI2-AS3 DAPK1-IT1
DB00755	Tretinoin	GPRC5A	approved; investigational; nutraceutical	C20H28O2	Leukemia	MIR17HG MALAT1 MAGI2-AS3 DAPK1-IT1
DB04216	Quercetin	STK17B	experimental; investigational	C15H10O7	Obesity	MIR17HG MALAT1 MAGI2-AS3
DB01254	Dasatinib	KIT	approved; investigational	C22H26ClN7O2S	Mast cell leukemia	MIR17HG MALAT1 MAGI2-AS3 CASC2
DB00583	Levocarnitine	CPT1A	approved; investigational	C7H15NO3	Nutritional deficiency	MIR17HG MALAT1 DAPK1-IT1
DB04552	Niflumic Acid	PLA2G4A	experimental	C13H9F3N2O2	Rheumatoid arthritis; Dysmenorrhea	MIR17HG MALAT1 MAGI2-AS3
DB01016	Glyburide	KCNJ8	approved	C23H28ClN3O5S	Type 2 diabetes	MIR17HG MALAT1 MAGI2-AS3

Tamoxifen is one of the selective estrogen receptor modulators, which is mainly used to treat estrogen receptor-positive breast cancer. In vitro study showed that tamoxifen-induced neutrophil apoptosis and efferocytosis by alveolar macrophages, thus improved lung inflammation [41]. And another study of peripheral blood and BALF in horses indicated tamoxifen might induce apoptosis in granulocytic cells [42]. Ruxolitinib is a Janus-associated kinase inhibitor indicated to treat bone marrow cancer. In asthmatic mice model, the ruxolitinib intervention suppressed Th17 cells’ survival through inhibiting JAK/STAT5 signaling pathway, promoted Th17 cells into apoptotic pathway by decreasing Bcl-2 and increasing Caspase3 expression [43]. Tretinoin, also known as all-trans-retinoic acid (ATRA), is used in the treatment of acute promyelocytic leukemia (APL). Wu et al. demonstrated ATRA decreased Th2 cytokines by inhibiting GATA-3 and increasing T-bet expression, also, ATRA down-regulated the expression of RORγt, thus decreased the level of IL-17A and the downstream proinflammatory factors [44]. Quercetin is a flavonol widely distributed in plants and is used to treat obesity. Pretreatment of quercetin was identified to inhibit gene expression of proinflammatory cytokine (TNF-a, IL-1b, IL-6, and IL-8) by regulating the activation of NF-κB and p38 MAPK [45]. Dasatinib, Levocarnitine, Niflumic Acid and Glyburide were also been validated to be beneficial in asthma improvement [46–49]. On the other hand, the results of drug repositioning indicated that lncRNAs which linked to drug-associated mRNAs were closely related to asthma.

## Discussion

Asthma is a heterogeneous disease, usually characterized by chronic airway inflammation. It is one of the most common chronic diseases and has been an economic burden all around the world [50]. In recent years, great efforts were done on discovering the new therapeutic targets and biomarkers of asthma, but most of the researches were focused on the protein-coding gene and small non-coding gene, the exploration of asthma-related lncRNA is still in its infancy. For diseases like asthma that do not use pathology tests to diagnose, providing a molecular marker for early screening with body fluids will be a convenient diagnostic method. The lncRNA in blood, urine and other body fluids are stable and tissue-specific, making it the most suitable molecular marker for early diagnosis and prognosis monitoring of diseases.

During the present study, we identified differentially expressed lnRNAs, mRNAs according to data from GEO and GENCODE database. Then by utilizing bioinformatics tools, we constructed a lncRNA-miRNA-mRNA ceRNA network, from which we extracted asthma related DE ceRNA network and recognized 5 potential key lncRNAs (MALAT1, MIR17HG, CASC2, MAGI2-AS3 and DAPK1-IT1). Further more, we performed literature validation and functional enrichment analysis, which helped proved that these lncRNAs were closely related to asthma from the side aspect.

Among the 5 key lncRNAs, MALAT1 and MIR17HG were thought to be most crucial as they linked to more verified asthma-related miRNAs and mRNAs, also, the functional enrichment results were much abundant than the other 3 lncRNAs. Although MALAT1 is well-known as a tumor-associated lncRNA [51], it also involved in inflammatory progression. Recently, more and more studies had explored its function in inflammatory progression. A study reported that MALAT1 regulated polarization of macrophage and response to lung injury [52]. Another study revealed MALAT1 regulated T-cell differentiation towards a Tregs phenotype, played an anti-inflammatory role in autoimmune neuroinflammation [53]. MIR17HG or the miR-17/92 cluster host gene was identified in 2004 [54]. The miR-17/92 cluster consists of six miRNAs: miR-17, miR-18a, miR-19a, miR-19b-1, miR-20a, and miR-92a-1. Carraro et al. had found that the miR-17/92 cluster was involved in normal lung morphogenesis, epithelial proliferation, and branching by targeting STAT3 and MAPK14 [55]. And the components of mir-17/92 cluster were also found to play roles in asthma. In a study of human bronchial epithelial cells (BEC), the expression of miR-19a was upregulated in severe asthmatics, which enhanced cell proliferation of BEC by targeting TGF-β receptor 2, the repression of miR-19a expression increased SMAD3 and decreased BEC proliferation [56].

For lncRNA CASC2, MAGI2-AS3 and DAPK1-IT1, probably because of the lack of current study, resulting in fewer analytical results, but they still have great potential to explore.

Clinically, the most effective way to treat and prevent exacerbation of asthma is drug therapy. At present, asthma drugs are mainly divided into two parts, bronchodilators and anti-inflammatory drugs. Although the current drugs are effective in treating most asthma patients, there is still a part of patients to whom the drugs are not working. In search of new drugs for asthma treatment, we performed drug repositioning by using the data obtained from DrugBank and TTD. And we got 8 drugs: Tamoxifen, Ruxolitinib, Tretinoin, Quercetin, Dasatinib, Levocarnitine, Niflumic Acid, and Glyburide. Although these drugs have been reported to be effectively improve asthma related symptoms and inflammatory process, there is still a distance from clinical application, further research should be done regarding drug dosage, administration methods, and dosage related side effects, etc.

## Conclusions

In summary, by constructing asthma related ceRNA network and conducting bioinformatic analysis, we identified 5 asthma associated key lncRNAs (MALAT1, MIR17HG, CASC2, MAGI2-AS3, DAPK1-IT1) and 8 potential new drugs (Tamoxifen, Ruxolitinib, Tretinoin, Quercetin, Dasatinib, Levocarnitine, Niflumic Acid, Glyburide). The current findings provide novel insights into the new therapeutic target and prognostic biomarker for asthma, and future work is needed to validate the key lncRNAs and their function in the ceRNA network.

## Data Availability

The dataset used and/or analyzed during the current study is available in the GEO repository, https://www.doi.org.10.1038/mi.2014.6 [13].

## Abbreviations

AHR: Airway hyperresponsiveness
ASMCs: Airway smooth muscle cells
ATRA: All-trans-retinoic acid
BALF: Bronchoalveolar lavage fluid
BEC: Bronchial epithelial cells
BLAST: Basic Local Alignment Search Tool
BP: Biological process
CC: Cell composition
ceRNA: Competing endogenous RNA
DE ceRNA network: Subnetwork related to DE mRNA and DE lncRNA
DE lncRNAs: Differentially expressed lncRNAs
DE mRNAs: Differentially expressed mRNAs
GEO: Gene Expression Omnibus
HDM: House dust mite
IGF-IR: Insulin-like growth factor-I receptor
IL-17RA: IL-17 receptor A
IPF: Idiopathic pulmonary fibrosis
KEGG: Kyoto Encyclopedia of Genes and Genomes
lncRNA: Long non-coding RNA
MF: Molecular function
miRNA: MicroRNA
MREs: MiRNA response elements
NSCLC: Non-small cell lung cancer
SAM: Significance analysis of microarrays
siRNA: Short interfering RNA
TTD: Therapeutic Target Database
